# EHMTI-0383. Spontaneous intracranial hypotension due to calcified micro-spurs perforating the dura – a case series

**DOI:** 10.1186/1129-2377-15-S1-J17

**Published:** 2014-09-18

**Authors:** J Beck, CT Ulrich, J Gralla, C Fung, J Fichtner, W Z'Graggen, A Raabe

**Affiliations:** 1 Neurosurgery, Inselspital, Bern, Switzerland; 2 Neuroradiology, Inselspital, Bern, Switzerland; 3 Neurology, Inselspital, Bern, Switzerland

## Introduction

Spontaneous intracranial hypotension (SIH) is a rare but increasingly diagnosed condition. Low cerebrospinal fluid (CSF) pressure due to a dural leakage causes orthostatic headache. The nature, etiology, and location of the CSF leak itself are currently unknown and are thought to be spontaneous or idiopathic in most cases.

## Aim

We present a case series with proven CSF leaks in which a systematic and meticulous search revealed dura-perforating micro-spurs as a cause of SIH.

## Methods

A consecutive series of patients with symptoms of intracranial hypotension were evaluated for a systematic diagnostic work-up from February to August 2013. We performed a spine focused, stepwise escalating imaging set. If a CSF leak could be restricted, we performed microsurgical exploration under intraoperative electrophysiological monitoring of the presumed site of the CSF-leak.

## Results

We identified 6 patients with SIH and imaging signs of spinal dural perforation. In 5 cases a calcified micro-spur extruding out of the disc space was identified perforated the dura and arachnoid and was the cause of CSF leak in all 5 cases. In one case a broad osteophyte had ripped the dura. All 5 micro spurs and one osteophyte were micro-surgically removed, the dura was sealed, and the CSF leak ceased immediately.

## Conclusions

The etiology of the CSF leak in SIH remains obscure. Here we present 6 patients in which a systematic spinal work-up, including microsurgical exploration, revealed dura perforating micro spurs. The dura-perforating micro spurs were a frequent, definitive, and readily treatable cause of SIH.

No conflict of interest.

